# Ancient and Recent Hybridization in the *Oreochromis* Cichlid Fishes

**DOI:** 10.1093/molbev/msae116

**Published:** 2024-06-12

**Authors:** Adam G Ciezarek, Tarang K Mehta, Angela Man, Antonia G P Ford, Geraldine Dorcas Kavembe, Nasser Kasozi, Benjamin P Ngatunga, Asilatu H Shechonge, Rashid Tamatamah, Dorothy Wanja Nyingi, Avner Cnaani, Titus C Ndiwa, Federica Di Palma, George F Turner, Martin J Genner, Wilfried Haerty

**Affiliations:** Earlham Institute, Norwich Research Park, Norwich NR4 7UZ, UK; Centre of Environment, Fisheries and Aquaculture Science (Cefas), Scientific Advice for Fisheries Management Team (SAFM), Lowestoft NR33 0H5, UK; Earlham Institute, Norwich Research Park, Norwich NR4 7UZ, UK; Earlham Institute, Norwich Research Park, Norwich NR4 7UZ, UK; School of Life and Health Sciences, Whitelands College, University of Roehampton, London SW15 4NA, UK; Department of Life Sciences, South Eastern Kenya University, Kitui 90200, Kenya; National Agricultural Research Organisation, Buginyanya Zonal Agricultural Research and Development Institute, Mbale, Uganda; Tanzania Fisheries Research Institute, Dar es Salaam, Tanzania; Tanzania Fisheries Research Institute, Dar es Salaam, Tanzania; Tanzania Fisheries Research Institute, Dar es Salaam, Tanzania; Ichthyology Section, National Museums of Kenya, Nairobi 00100, Kenya; Institute of Animal Science, Agricultural Research Organization, Rishon LeZion 7528809, Israel; Department of Clinical Studies, University of Nairobi, Nairobi, Kenya; School of Biological Sciences, University of East Anglia, Norwich NR4 7TU, UK; School of Natural Sciences, Bangor University, Bangor LL57 2UW, UK; School of Biological Sciences, University of Bristol, Bristol BS8 1TQ, UK; Earlham Institute, Norwich Research Park, Norwich NR4 7UZ, UK

**Keywords:** hybridizations, *Oreochromis*, cichlid, Phylogenomics, introgression, speciation

## Abstract

Cichlid fishes of the genus *Oreochromis* (tilapia) are among the most important fish for inland capture fisheries and global aquaculture. Deliberate introductions of non-native species for fisheries improvement and accidental escapees from farms have resulted in admixture with indigenous species. Such hybridization may be detrimental to native biodiversity, potentially leading to genomic homogenization of populations and the loss of important genetic material associated with local adaptation. By contrast, introgression may fuel diversification when combined with ecological opportunity, by supplying novel genetic combinations. To date, the role of introgression in the evolutionary history of tilapia has not been explored. Here we studied both ancient and recent hybridization in tilapia, using whole genome resequencing of 575 individuals from 23 species. We focused on Tanzania, a natural hotspot of tilapia diversity, and a country where hybridization between exotic and native species in the natural environment has been previously reported. We reconstruct the first genome-scale phylogeny of the genus and reveal prevalent ancient gene flow across the *Oreochromis* phylogeny. This has likely resulted in the hybrid speciation of one species, *O. chungruruensis*. We identify multiple cases of recent hybridization between native and introduced species in the wild, linked to the use of non-native species in both capture fisheries improvement and aquaculture. This has potential implications for both conservation of wild populations and the development of the global tilapia aquaculture industry.

## Introduction

To meet the food demands of a growing human population, global aquaculture production has increased dramatically in recent decades, and is projected to play a key role in alleviating nutritional poverty in the coming decades (Hall et al. 2017; Ahmed et al. 2019; Naylor et al. 2021). Tilapia of the genus *Oreochromis* (Cichlidae: Oreochromini) are a group of cichlid fishes native to Africa and the Middle East, and are now the second largest group (by tonnage produced) of any aquaculture fish globally, following carp (Cyprinidae). In addition to aquaculture, *Oreochromis* support important regional capture fisheries. Although programs to enhance fisheries production in East Africa have been in place since the 1950s, yields have stagnated in recent decades (Shechonge et al. 2019). *Oreochromis* aquaculture production is dominated by a single species, the Nile tilapia, *Oreochromis niloticus*, which accounted for 4.5 out of the total 5.5 million tons of tilapia produced in 2020 (FAO 2022). The predominance of Nile tilapia has been attributed to high growth rates as well as tolerance of a wide range of environmental conditions, including high temperature and low dissolved oxygen (El-Sayed and Fitzsimmons 2023). Wild relatives of the Nile tilapia, many of which support their own important fisheries, in selective breeding programs could be used to further enhance production by introducing variants associated with key aquaculture traits (Lind et al. 2012). However, despite this importance for food security, many of the 37 described *Oreochromis* species (Ford et al. 2019) are poorly known.

Given the high productivity of some *Oreochromis* species in aquaculture and capture fisheries, they have been widely introduced across tropical and subtropical freshwaters globally (Lévêque 1995; Canonico et al. 2005). In Africa, introductions to the natural environment have resulted in widely documented recent hybridization (Shechonge et al. 2018; Bradbeer et al. 2019; Blackwell et al. 2020; Champneys et al. 2021; Ciezarek et al. 2022). In particular, hybridization with *O. niloticus* has been shown to present conservation risks to native tilapia species (Deines et al. 2014), although the long-term fitness implications of admixture are generally unknown. The likelihood of hybridization between native and exotic *Oreochromis* species is likely to increase with the projected growth of aquaculture across Africa (Prabu et al. 2019), particularly as aquaculture facilities have been demonstrated to drive colonization of invasive *O. niloticus* populations to proximate water bodies (Forneck et al. 2021). Detailed analysis of the extent of hybridization between co-occurring tilapia species will therefore be valuable for identifying species and populations of potential conservation concern.

Hybridization of ancestral lineages, resulting in genomic introgression, markedly influences the evolutionary history of lineages. To date, analysis of ancestral introgression during the evolutionary history of *Oreochromis* has been hampered by a lack of genomic data for much of the group. Characterizing the degree of ancestral introgression may allow better understanding of potential risks and consequences of modern hybridization, for example by examining the capability of different species to hybridize, or the extent to which it may drive radiation. Considerable attention has been given to a closely related group of cichlid fishes: the haplochromines, which diverged from tilapia approximately 20 Mya (Kumar et al. 2017). It has been proposed that the explosive African crater lake radiations of this group have been facilitated by the introgression arising from hybridization of ancestral lineages (Meier et al. 2017; Svardal et al. 2020). Specifically, it has been suggested that differentiation and diversification have been fueled by novel combinations of genetic variants (Singhal et al. 2021) as well as sharing of ancient alleles that were already filtered by selection (Marques et al. 2019; Meier et al. 2019). This may have driven rapid speciation and extensive ecomorphological diversification, despite low levels of genetic diversity (Malinsky et al. 2018; McGee et al. 2020; Svardal et al. 2021). Hybridization between weakly diverged lineages may also enable diversification by generating novel trait combinations (Seehausen 2004). By contrast, introgression, ongoing or recurring hybridization may also decrease differentiation and homogenize gene pools, therefore reducing the propensity of a lineage to radiate. The relationship between hybridization, introgression, and diversification is therefore complex.

In contrast to the haplochromines, extensive radiation has not taken place in *Oreochromis* (Seehausen 2006). The only known *Oreochromis* lacustrine radiations are small and limited to Lake Malawi and Lake Natron (3 species each). Most *Oreochromis* species are allopatric to apparent sister taxa (Trewavas 1983; Ford et al. 2019). To date, comprehensive phylogenomic examinations of ancestral introgression occurring during the evolution of *Oreochromis* are lacking. It is currently unclear what, if any, role ancestral introgression has played in *Oreochromis* diversification.

This study focuses on the genomic implications of ancestral and recent introgression in *Oreochromis*, focusing on wild populations in Tanzania. The country is a hotspot for diversity of the genus with at least 21 species present, including 3 species that have been widely introduced outside their native range, namely *O. niloticus*, *O. leucostictus*, and *O. esculentus* (Shechonge et al. 2019). To reconstruct the phylogenetic relationships between species, and to quantify ancestral and modern hybridization, we present a genome-wide resequencing dataset of the genus *Oreochromis*. We provide a first phylogenomic analysis of the group and show that *Oreochromis* is typified by a high degree of ancestral introgression. We also identify a species of hybrid origin, the Lake Kiungululu tilapia, *O. chungruruensis*. We further confirm that anthropogenic activities have resulted in widespread recent hybridization, highlighting a potential risk to the conservation of species and their genetic diversity.

## Materials and Methods

### Sampling and Sequencing

**Fig. 1.**
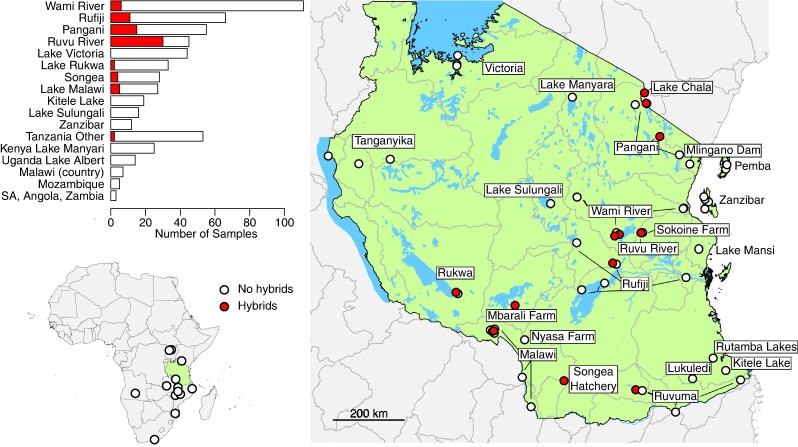
Locations of the *Oreochromis* sampled across Africa (bottom left) and, within Tanzania (right). Sample sites where recent hybrids were identified from genomic data are shown with filled circles. Key drainage basins are labeled. The bar chart (top left) shows the total number of specimens (recent hybrids in filled bar, non-hybrids in unfilled bar) located per drainage basin within Tanzania, for all basins with more than ten samples, or countries outside of Tanzania. All basins within Tanzania with fewer than ten specimens sampled are grouped into “Tanzania Other.” Abbreviation: SA, South Africa. Geographic coordinates of sampling locations are shown in supplementary table S1, Supplementary Material online.

We sampled and generated sequencing data for 433 individuals and combined this with previously published data from 142 individuals (supplementary table S1, Supplementary Material online) from across southern and eastern Africa (Fig. 1). Samples were from 65 locations, 21 drainage basins across Tanzania, as well as South Africa, Kenya, Angola, Malawi, Mozambique, and Uganda. Individuals were morphologically identified to species using information outlined by Genner et al. (2018). Individuals were classified as hybrids if they had characteristics intermediate between different species. Our final dataset comprised of 25 species, including the outgroups *Maylandia zebra* and *Sarotherodon galilaeus* and 23 out of the 37 species of *Oreochromis* currently recognized, including *Oreochromis (Alcolapia) grahami* (Fricke et al. 2023). *Maylandia zebra* is a haplochromine cichlid native to Lake Malawi, estimated to have diverged from *Oreochromis* 14.1 to 30 million years ago. *Sarotherodon galilaeus* is a widely distributed cichlid fish, found across Northern Africa and the Middle East, estimated to have diverged from *Oreochromis* 14.1 million years ago (Kumar et al. 2017). Fish specimens were mostly obtained from local fishers or through survey fishing. Live fish were euthanized with an overdose of anesthetic (MS-222). Samples for subsequent DNA extraction were collected by clipping off the right pectoral fin, which was placed in a labeled vial of ethanol. Whole voucher specimens (currently stored in research collections at Bangor University and University of Bristol) were retained, with each individual pinned to a Styrofoam board, photographed, and preserved in formalin or ethanol.

DNA was extracted from fin clips using a PureLink Genomic DNA extraction kit (Life Technologies). Sequencing was conducted using either automated KAPA DNA library preparation with Illumina HiSeq 2500 (125 bp paired-end), LITE library preparation with Illumina HiSeq 4000 (150 bp paired-end), or LITE library preparation with NovaSeq S4 (150 bp paired-end).

### SNP Calling

SNPs were called against previously published near chromosome-level assemblies of *M. zebra* (GCF_000238955.4) and *O. niloticus* (GCF_001858045.2) (Conte et al. 2019). Raw reads were checked for adapter sequence contamination using BBMerge, within BBMap (v38.06) (Bushnell 2014). Where necessary, reads were trimmed for adapters using fastp (v0.20.0) (Chen et al. 2018), with quality trimming disabled. Reads were then mapped separately against both assemblies using the mem function in bwa (v0.30.0) (Li and Durbin 2009), with raw mappings sorted by coordinate and having mate coordinates and insert size fields added using samtools (v1.9) (Li et al. 2009). Joint genotyping was then carried out on all 575 samples using bcftools (v1.10.2) (Li et al. 2009). First, bcftools mpileup was used with minimum base and mapping qualities of 30. Multi-allelic variant calling was then carried out using bcftools call. Variants were filtered (the full site excluded from the dataset) if they were within three base pairs of any other variant, if variant quality score was less than 30, if depth at a given site (across all samples) was less than 500 or greater than 9,000 (∼one-third the average sequencing depth for half of samples, or more than 3 × the average sequencing depth), or if minor allele count was less than 3 (to ensure SNPs were present in more than one individual, reducing the likelihood of genotyping error).

We tested for batch effects for different sequencing methods using all *O. niloticus* samples as follows (using the *M. zebra* mapping). Genotype likelihoods were estimated for each individual using angsd (v0.923) (Korneliussen et al. 2014), with a minimum SNP *P*-value of 1 × 10^−6^ and minimum mapping quality of 30. Linkage disequilibrium was then calculated between sites using ngsLD (v1.2.1) (Fox et al. 2019), with sites with *r*^2^ > 0.6 over 20 kb windows filtered to reduce linkage. A principal component analysis (PCA) was then calculated from these genotype likelihoods using PCAngsd (v1.2.1) (Meisner and Albrechtsen 2018).

### Backbone Phylogeny Reconstruction

To construct a species-level phylogeny, we identified a panel of 91 reference individuals that are less likely to be recent hybrids, given potential recent gene flow (supplementary table S1, Supplementary Material online). These individuals were identified using morphology, evidence of monophyly in a full genome neighbor-joining tree of individuals not classified morphologically as hybrid, and evidence of monophyly in a mitochondrial genome neighbor-joining tree of those individuals (supplementary data S1, Supplementary Material online). Using this reference panel, separate phylogenetic trees were inferred for each of the *M. zebra* and *O. niloticus* mapped datasets. Trees were constructed using both a maximum-likelihood SNP-based concatenation tree, inferred using IQ-TREE (v1.6.12) (Nguyen et al. 2015), as well as a multi-species coalescent summary-based tree, inferred using ASTRAL (v5.4.12) (Zhang et al. 2018; Rabiee et al. 2019), based on trees inferred from recombination-free 10 kb windows across the genome with IQ-TREE (supplementary methods, Supplementary Material online). Robinson–Foulds (RF) distances (Robinson and Foulds 1981), which are twice the number of bipartitions divided by internal branches discordant between two topologies, were calculated between each 10 kb window tree and the inferred ASTRAL species tree using the ETE 3 Python package (Huerta-Cepas et al. 2016), in order to further quantify how widespread phylogenetic discordance was. For species where the inferred phylogenetic trees raised taxonomic questions, such as a lack of monophyly of all individuals assigned to a species, exploratory PCA was carried out on all individuals of the relevant species, but excluding those inferred to be a recent hybrid (see below), using PLINK (v2.0.0) (Purcell et al. 2007). The PCAs on each subset of individuals used all biallelic SNPs found with a minor allele count of at least 3 in this subset of individuals, and SNPs were pruned for linkage disequilibrium (*r*^2^ > 0.6) over 20 kb windows using bcftools.

### Assessment of Ancestral Introgression

Ancestral introgression between species was assessed using the panel of 91 reference individuals, and both the *M. zebra* and *O. niloticus* mapping datasets. We used both SNP-based D statistics (Green et al. 2010), *f*-branch statistics (Malinsky et al. 2018), calculated using Dsuite (v0.4) (Malinsky et al. 2021) and Dp statistics (Hamlin et al. 2020) and genome-wide 10 kb window phylogenetic tree-based analyses. These tree-based analyses included the branch-length tests (BLT) and discordant count tests (DCT), using the scripts provided by Suvorov et al. (2022), and chi-squared tests of discordant topology frequencies inferred using IQ-TREE (Minh et al. 2020; Suvorov et al. 2022) (supplementary methods, Supplementary Material online). To test whether introgression was more likely between species sharing a drainage basin or between closely or more distantly related species, for each species we identified the species counterpart with the highest *f*-branch (i.e. the highest degree of gene flow). Internal nodes were not considered for this analysis to ensure we were only comparing species pairs. We then inferred whether these species pairs were more likely to occupy the same modern drainage basins than expected by chance using a randomized permutation test. A simulated distribution of counts of species counterparts with shared drainage basins was generated. For each species, a random counterpart was chosen, considering the probability for each that it shared a drainage basin. The total sharing drainage basin was then recorded at the end of each of 100,000 simulations, and the proportion of simulations with at least the observed number given as a *P*-value. Similarly, we identified for each species whether their high *f-*branch was more or less phylogenetically related than the average of all the species compared (*z* ≤ −2 for less phylogenetically distant, *z* ≥ 2 for more phylogenetically distant). We then tested whether we observed more species with significantly phylogenetically similar or dissimilar counterparts (compared to the mean phylogenetic distance, measured by coalescent distance on the ASTRAL tree used for *f*-branch analysis) than expected by chance using a permutation test. Simulations for this were carried out as for the drainage basin analysis, except counting those significantly more or less distantly related. When a pair of species matched each other as its highest *f*-branch counterpart, only one (chosen at random) was utilized in the permutation tests to avoid pseudo-replication of a single introgression event. This was repeated ten times, with a different random choice each time, with the highest *P*-value recorded.

### Analysis of Putative Introgression Events

Putative introgression events, identified by the *f-*branch analysis, were further investigated to identify introgressed genomic regions. This was carried out using a novel statistic (Dwt; Dweighted_topo), derived from topology weightings (a quantification of the relative contribution of each individual topology to the full tree) calculated from “topology weighting by iterative sampling of subtrees” (Twisst) (Martin and Van Belleghem 2017), computed along the 22 linkage groups of the *M. zebra* reference assembly (supplementary methods, Supplementary Material online). Dwt is designed to highlight putatively introgressed regions. Dwt = 0 indicates regions where the species tree (sptree) has the highest weighting. Dwt > 0 indicates where disc1 (the putative introgression topology) is most heavily weighted, with one indicating it is fully supported (no weighting for either of the other topologies). Similarly, Dwt < 0 indicates where disc2 (the other alternate topology) is most heavily weighted.


$$Dwt\;=\frac{disc1\;-\;disc2}{disc1\;\;+\;\;disc2}*\frac{max(sptree,\;disc1,\;disc2)-sptree}{max(sptree,\;disc1,\;disc2)\;+sptree}$$

### Tests of Hybrid Speciation

Putative introgression events where the Twisst analysis showed no clear excess of either the species tree topology or hybridization topology were further investigated to identify signals of hybrid speciation. Following Olave et al. (2022), we used ADMIXTURE analyses of the relevant species at both K = 2 and K = 3, as well as assessment of species-diagnostic SNPs. Putative introgression dates were also estimated based on estimates of Dxy and π in phylogenetically identified introgressed regions of the genome, calculated using genomics_general (https://github.com/simonhmartin/genomics_general) (see supplementary methods, Supplementary Material online).

### Recent Hybridization

The 486 individuals who were not among the reference panel were tested for evidence of recent hybridization. We carried out separate analyses in each sampled water drainage basin within Tanzania. For each drainage basin, biallelic SNPs were extracted for each sampled individual, alongside reference individuals for all the taxa recorded in the water body by Shechonge et al. (2019). SNPs with a minor allele count of at least 3 were retained, and SNPs were pruned for linkage disequilibrium (*r*^2^ > 0.6) over 20 kb windows using bcftools. For each drainage basin, a supervised ADMIXTURE (Alexander et al. 2009) analysis was carried out only involving the relevant species or populations, with a K value equal to the number of locally recorded taxa for the ADMIXTURE analysis, and each reference species assigned to their known group. Test individuals were assigned a hybrid status if they had multiple ancestry components where the lower end of the standard error was at least 0.1. This cutoff was used to identify up to second-generation backcrosses, although we note other more complicated hybridization histories could result in elevated ancestry components. We further confirmed that all the reference individuals of the same species were tightly clustered using PCAs performed in PLINK, and comprised a monophyletic group with no long branch lengths, in phylogenetic trees inferred with IQ-TREE (v2.0) with automated model detection, 5 independent runs and 1,000 rapid bootstraps. These analyses were carried out with the same SNP set as the ADMIXTURE runs, except it was further filtered for sites where at least one individual had each of the homozygous reference and alternate alleles for the IQ-TREE analysis. We further characterized recent hybridization using panels of species-diagnostic SNPs (supplementary methods, Supplementary Material online).

## Results

Mapping, phylogenomic, and ancestral introgression analyses were carried out against both the *O. niloticus* and *M. zebra* reference assemblies separately. Results were similar and conclusions identical (supplementary materials, Supplementary Material online), therefore only the *M. zebra* reference analyses are reported in the following.

### Sampling, Sequencing, Read Mapping, and SNP Calling of 23 Species

An average of 41.3 million paired-end reads were sequenced for the 433 individuals (range 10.4 to 106 million) (supplementary table S1, Supplementary Material online). Mapped reads from all 575 individuals (including the previously published data for 142 individuals) had average depth of 6.3 (range 1.7 to 18.6), and average properly paired mapping of 75% (range 45% to 89%). In total 55,949,298 filtered SNPs were called. Full mitochondrial genomes of at least 10,000 bp were successfully assembled for 437 out of the 450 individuals not morphologically identified as hybrid, with an average length of 16,541 bp (range 11,048 to 17,126 bp). No batch effect was apparent, with *O. niloticus* samples clustering with geographic location, rather than library preparation method (supplementary fig. S1, Supplementary Material online).

### Phylogenetic Inference

**Fig. 2.**
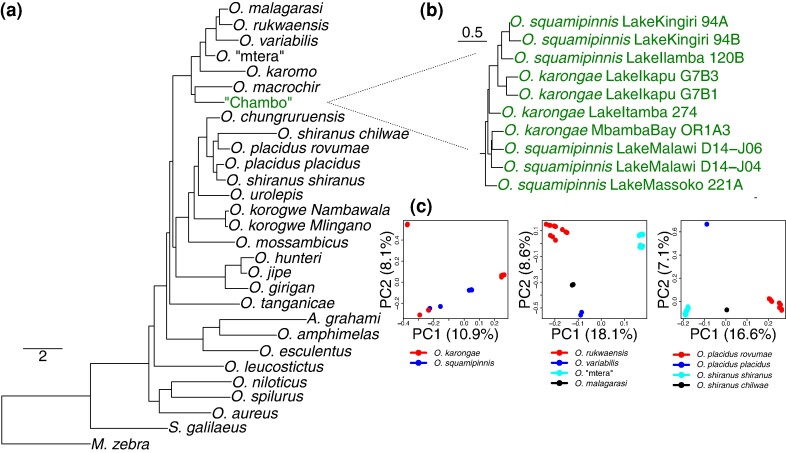
a) Phylogenetic tree inferred using ASTRAL from the *M. zebra* mapping dataset, with conspecific individuals collapsed. Branch lengths are in coalescent units. All nodes had posterior probability of 1.0. b) Phylogenetic tree inferred using ASTRAL from the *M. zebra* mapping dataset, without species collapsed, highlighting only the individuals from the Lake Malawi “Chambo” assemblage of *O. squamipinnis* and *O. karongae*. c) PCA analyses of the major taxonomic questions raised by this phylogenetic tree, showing the “Chambo” group (left), *O. rukwaensis* being distinct from *O. “*mtera” (middle), and subspecies of *O. placidus* and *O. shiranus* not supporting current classification (right).

A total of 1,692,136 non-coding SNPs were used for the maximum-likelihood phylogenetic inference with 15,310 recombination-free (see supplementary methods, Supplementary Material online) 10 kb windows used for ASTRAL. Most of the taxonomic species produced monophyletic groups, and therefore collapsed to a single node. Those that were not monophyletic were instead collapsed to the population level (Fig. 2a). There were some inconsistencies between the ASTRAL and maximum-likelihood trees, particularly regarding the phylogenetic placement of *O. tanganicae, O. placidus placidus*, and *O. korogwe* (supplementary figs. S2 to S5, Supplementary Material online). The ASTRAL results also suggest *O. hunteri* to be a monophyletic species, whereas the ML trees do not (supplementary figs. S2 to S5, Supplementary Material online). All following analyses are reported using the ASTRAL species tree, given that unlike the maximum-likelihood tree it accounts for the likely widespread incomplete lineage sorting in the dataset. This is indicated by the high levels of phylogenetic discordance shown by high RF distances between each 10 kb window tree and the species tree. No 10 kb window tree had an RF distance from the species tree of less than 0.08 (supplementary fig. S6, Supplementary Material online).

There were instances of taxonomic species where individuals from different populations did not form monophyletic groups (Fig. 2a, supplementary fig. S2, Supplementary Material online), and PCA analyses further confirmed a lack of clustering of taxonomically identified species (Fig. 2c). *O. rukwaensis* individuals from the Rukwa drainage basin were resolved as a clade but were phylogenetically distinct from individuals collected from the Mtera Reservoir, which had provisionally been identified as *O. rukwaensis* based on morphology (Shechonge et al. 2019). Here, we refer to this population as *O.* “mtera”. We found no evidence of a taxonomic split between *O. shiranus* and *O. placidus*, and instead *O. shiranus shiranus* was resolved as sister to *O. placidus placidus*, whereas *O. shiranus chilwae* is sister to *O. placidus rovumae*. Similarly, the analyzed “Chambo” species, *O. squamipinnis*, and *O. karongae*, are not reciprocally monophyletic in our analyses (Fig. 2).

### Widespread Ancestral Introgression

There was consistently a high degree of ancestral introgression across the *Oreochromis* phylogeny evident in our analyses. Using the phylogenetic trees constructed from the 15,310 10 kb recombination-free windows, 44,030 out of the 113,564 trios tested with the BLT and DCT tests showed significant deviations from expected values under incomplete lineage sorting alone, consistent with introgression. Similarly, the frequencies of 10 kb window tree discordant topologies indicated introgression at 42/89 nodes in the ASTRAL tree. For the SNP-based D statistics, 2,658 out of the 2,925 tested trios showed significantly higher levels of allele sharing between non-sister taxa than expected, with Dp values (Hamlin et al. 2020) indicating a range of introgression proportions ranging from 0 to 0.45 (supplementary table S2, Supplementary Material online; for this analysis, individuals were pooled into populations or species, hence the lower number of tested trios than the BLT and DCT analyses).

**Fig. 3.**
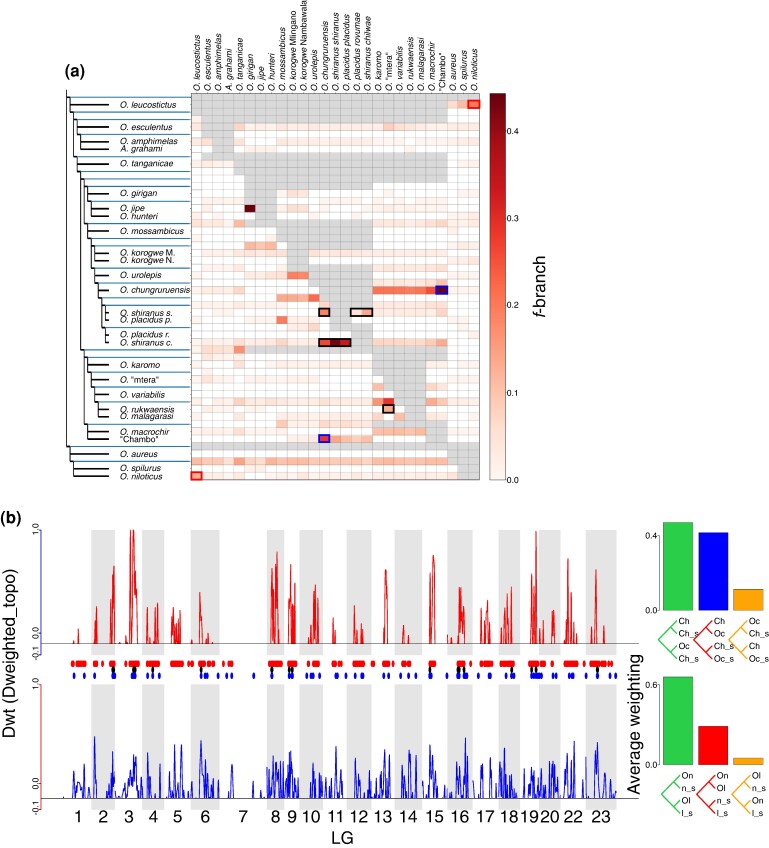
a) Heatmap of the *f*-branch values calculated between each node (*y* axis) and tip (*x* axis) of the *M. zebra* mapping ASTRAL tree. Boxes indicating the introgression from “Chambo” into *O. chungruruensis* are highlighted in blue, and between *O. leucostictus* and *O. niloticus* are highlighted in red. Boxes indicating introgression amongst the clades associated with taxonomic uncertainties are highlighted in black. b) Dwt statistics across the linkage groups showing regions with a stronger weighting for the introgression tree (above the solid line), where *O. leucostictus* and *O. niloticus* are sister (upper; red), and where “Chambo” and *O. chungruruensis* are sister (lower; blue). Dots represent stretches of the genome ≥50 kb where Twisst analyses consistently have Dwt = 1.0 showing introgression between *O. leucostictus* and *O. niloticus* (red), *O. squamipinnis* and *O. chungruruensis* (blue), and overlap between both comparisons (black). Abbreviations: On—*O. niloticus*, Ol—*O. leucostictus*, On_s—*O. spilurus* + *O. aureus*; Ol_s—*O. variabilis* + *O. esculentus* + *A. grahami,* Ch—“Chambo”, Oc—*O. chungrurensis*, Ch_s—*O. rukwaensis* + *O. variabilis* + *O. macrochir*, Oc_s—*O. placidus rovumae* + *O. korogwe* Mlingano + *O. mossambicus*.

F-branch statistics were calculated between each branch in the tree and each tip, revealing a few branches which appeared to exhibit relatively large amounts of gene flow (Fig. 3a). Notably high *f*-branch values were recorded associated with two of the taxonomic uncertainties highlighted above; namely the *O. shiranus—O. placidus* group (especially involving *O. shiranus chilwae*) and between *O. rukwaensis* and *O. “mtera”* (Fig. 3a).

We identified the counterpart for each species which had the highest *f*-branch value. Five counterparts (*O. chungruruensis* with *O. shiranus shiranus*, *O. leucostictus* with *O. niloticus*, *O. girigan* with *O. korogwe* Mlingano, *O. jipe* with *O. girigan*, and *O. karomo* with *O. tanganicae*) shared modern drainage basins. This was more than expected by chance (*P* = 0.003), but within the simulated range (0 to 7 species pairs). Similarly, the species pairs with evidence of introgression were more likely to be more closely related than the mean phylogenetic distance between each species (*Z*-score ≤ −2) than expected by chance (4 species; *P* = 0.0004), but within the simulated range (0 to 5). This was the case for four species pairs: *O. jipe* with *O. girigan*, *O. amphimelas* with *O. esculentus*, *O. rukwaensis* with *O.* “mtera”, and *O. malagarasi* with *O. variabilis*.

Based on the results of *f-*branch analyses and Dp values, we focused on ancestral introgression between *O. niloticus* and *O. leucostictus* and between *O. chungruruensis* and the “Chambo” group. These were the species pairs indicating the highest proportions of introgression according to Dp (supplementary table S2, Supplementary Material online), as well as the highest *f-*branch values between non-sister or closely related branches (supplementary fig. S7, Supplementary Material online). Four analyses were carried out to assess gene flow in each case (supplementary materials, Supplementary Material online). All analyses from the Dweighted_topo statistics show generally similar putatively introgressed regions between the different analyses, although some species combinations suggested more than others (supplementary fig. S8, Supplementary Material online). In both cases, very little of the genome had a higher weighting for the other alternate topology (non-species or introgression tree), as would have been indicated by Dwt < 0 (Fig. 3b). In total, 52 MB of the genome was consistently found to be introgressed between *O. leucostictus* and *O. niloticus,* and 4 MB introgressed between *O. chungruensis* and “Chambo” (Fig. 3b), from 761 MB in the *M. zebra* reference genome linkage groups. There was significantly more of the genome introgressed in both comparisons (816 kB, with regions across all 22 linkage groups; Fig. 3b) than would be expected randomly (permutation test *P* = 0.0002; supplementary fig. S9, Supplementary Material online).

**Fig. 4.**
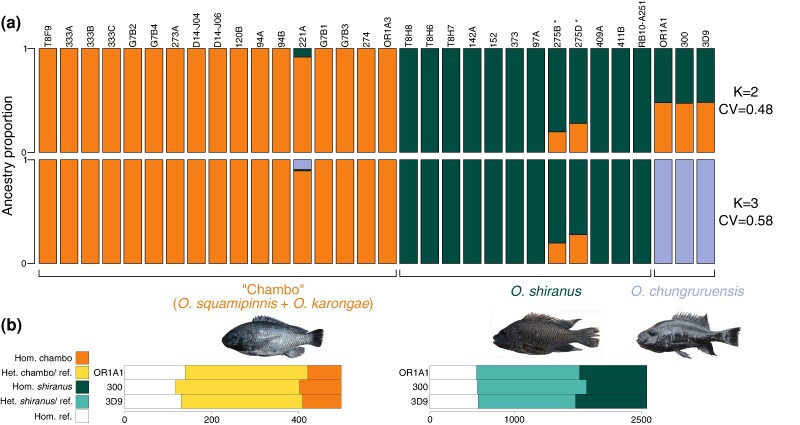
a) ADMIXTURE analysis of all *O. shiranus—O. placidus*, “Chambo”, and *O. chungruruensis* at *K* = 2 and *K* = 3. b) The proportion of species-diagnostic SNPs which are either homozygous for the alternate allele (Hom.), heterozygous (Het.), or homozygous for the reference allele (ref.) in the three *O. chungruruensis* individuals (abbreviation *shi-plac—O. shiranus + O. placidus*). *indicates genetically identified *O. shiranus shiranus* × “Chambo” hybrids in Lake Itamba.

**Fig. 5.**
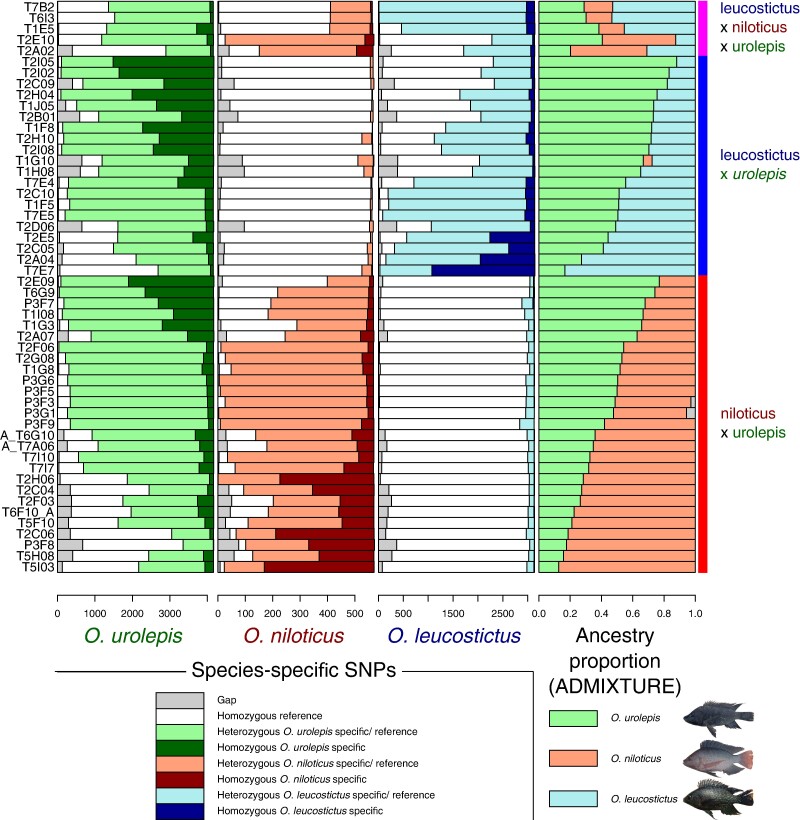
Species diagnostic SNP frequency for each individual inferred to be a hybrid between the potential parent species of *O. leucostictus*, *O. niloticus*, and *O. urolepis* from ADMIXTURE analyses (ancestry components on far-right) (one per row). Diagnostic SNPs shown for *O. urolepis* (left), *O. niloticus* (mid-left), and *O. leucostictus* (mid-right).

The nature of *O. chungruruensis* introgression with “Chambo” and *O. shiranus shiranus* was investigated by ADMIXTURE analysis assuming two populations (K = 2; cross-validation score = 0.48), with individuals of “Chambo,” *O. chungruruensis*, and *O. shiranus shiranus* from the Lake Malawi basin (*O. placidus* and *O. shiranus chilwae* were not recorded here so was not used). This suggested that 47% to 48% of the *O. chungruruensis* genome had affinity with “Chambo”, and 52% to 53% with *O. shiranus shiranus*. When assuming three populations (K = 3; cross-validation score = 0.58), *O. chungruruensis* formed its own group (Fig. 4). Diagnostic SNPs were also identified for “Chambo”, the *O. shiranus—O. placidus* group and *O. chungruruensis* (supplementary results, Supplementary Material online). Note that diagnostic SNPs could not be detected for *O. shiranus shiranus* alone, and so the monophyletic *O. shiranus—O. placidus* group was used as a whole. These showed similar frequencies of SNPs fixed for either the reference or the homozygous alternate alleles of both “Chambo” and *O. shiranus—O. placidus*, with around half of diagnostic SNPs heterozygous (Fig. 5). Across the 22 linkage groups, 4,300 windows (each of length 200 SNPs) were found where all haplotypes *O. chungruruensis* and *O. shiranus—O. placidus* were sister taxa, compared to 2,247 windows where *O. chungruruensis* and “Chambo” were sister. Based on values of π and Dxy within these windows, we inferred introgression times of 21,520 (CI 16,374 to 47,075) years ago between *O. shiranus/O. placidus* and *O. chungruruensis* and 13,565 (CI 10,321 to 29,673) years ago between “Chambo” and *O. chungruruensis*.

### Recent Hybridization Ongoing Across Tanzania

Eleven different drainage basins and three aquaculture establishments across Tanzania were tested for the presence of hybrids (Fig. 1). A total of 76 hybrids (Table 1) were identified. These were identified from across seven drainage basins: Ruvu River (30/46 tested individuals identified as hybrid), Pangani River (15/51), Rufiji River (11/56), Wami River (6/109), Lake Malawi (2/12), Lake Rukwa (2/29), Ruvuma River (1/3), and Lake Chala (1/1). Hybrids were also identified at two aquaculture establishments: the Songea hatchery (7/50) and Mbarali Farm (1/24). It should be stressed that these numbers should not be taken as representative, as sampling was non-random and biased toward individuals thought to be hybrids on the basis of morphology.

**Table 1:** Recent hybrids identified from the supervised ADMIXTURE analysis

Species pair	Number of hybrids	Drainage basins recorded (number; locations)
*O. niloticus × O. urolepis*	27	Ruvu River (10; Mindu Reservoir), Rufiji (9; Kidatu), Pangani (7; Lake Jipe), Wami River (1; Kilosa)
*O. leucostictus* × *O. urolepis*	20	Ruvu River (17; Mindu), Wami River (3; Kilosa (2), Lake Nala (1))
*O. niloticus × O. leucostictus* × *O. urolepis*	5	Ruvu River (3; Mindu), Wami River (2; Kilosa)
*O. niloticus × O. shiranus*	5	Songea hatchery (5)
*O. niloticus × O. “*mtera”	3	Rufiji (2; Kidatu), Mbarali Farm (1)
*O. shiranus ×* “Chambo”	2	Lake Malawi (2; Lake Itamba)
*O. leucostictus × O. shiranus*	2	Songea hatchery (2)
*O. girigan × O. jipe*	2	Pangani (2; Lake Jipe)
*O. girigan × O. niloticus*	2	Pangani (2; Lake Jipe)
*O. girigan × O. jipe × O. niloticus*	2	Pangani (2; Lake Jipe)
*O. girigan × O. esculentus*	2	Pangani (2; Lake Jipe)
*O. niloticus × O. placidus rovumae*	1	Ruvuma (1; Rovuma)
*O. leucostictus × O. rukwaensis*	1	Lake Rukwa (1; Lake Rukwa)
*O. esculentus × O. leucostictus*	1	Lake Rukwa (1; Lake Rukwa)
*O. rukwaensis × O. urolepis* ^a^	1	Lake Chala (1; Lake Chala)

See supplementary table S1, Supplementary Material online for exact locations and ancestry components of each hybrid.

^a^Indicates the individual with the “Bandia” phenotype with Lake Chala.

Most of the hybrids in our samples (68%) were between *O. urolepis, O. leucostictus*, and *O. niloticus*, with none directly between *O. leucostictus* and *O. niloticus*. Species-diagnostic SNPs, fixed in the confidently non-hybrid individuals of each of these three species, and with a low frequency (<0.1) in other non-hybrid individuals, were identified for each, with numbers ranging from 571 in *O. niloticus*, to 3,147 in *O. leucostictus* and 4,177 in *O. urolepis*. The hybrids identified by ADMIXTURE between the three species were investigated for these diagnostic SNPs, with results generally concordant with the ADMIXTURE ancestry components (Fig. 5). The individuals with ADMIXTURE ancestry components close to 50% had high levels of heterozygosity for both species, potentially indicating F1 hybrids. When ancestry components rose above 50% for a species, it was generally associated with more homozygous species-diagnostic SNPs, suggesting backcrossing.

Only 51/124 individuals morphologically suggested to be possible hybrids were genetically confirmed, ranging across the Rufiji, Ruvu River, Wami River, Zanzibar, as well as the Songea hatchery. 24 out of the remaining 321 test individuals were genetically, but not morphologically identified as hybrids. These included individuals morphologically assigned to *O. urolepis* from Mbarali Farm, the Ruvu River, and the Wami River, *O. shiranus shiranus* from Lake Itamba and Songea hatchey, *O. niloticus* from Lake Jipe*, O. girigan* from Lake Jipe, *O. esculentus* from Lake Rukwa, *O. rukwaensis* from Lake Rukwa, *O. pangani* from Lake Kalimau, and *O. placidus rovumae* from the Muhuwesi River (a tributary of the Ruvuma River).

The “Bandia” individual, from a potentially hybrid population found in Lake Chala of unknown origin, was identified as a probable *O. rukwaensis* × *O. urolepis* hybrid, according to a panel of species-diagnostic SNPs, as well as ADMIXTURE and Twisst analysis (see supplementary results, Supplementary Material online).

## Discussion

Here, we present evidence that the genus *Oreochromis*, a group of cichlid fish important for global aquaculture, has undergone introgressive hybridization multiple times during its evolution, and is now experiencing large-scale recent hybridization resulting from anthropogenic translocations. Almost all evidence of recent hybrids involved populations introduced to boost aquaculture or fisheries production.

### Phylogenomic Resolution of Oreochromis and Taxonomic Questions

We inferred a first phylogenomic backbone tree for the clade, building on previous work that was based on a handful of nuclear and mitochondrial markers (Ford et al. 2019). In accordance with Ford et al. (2019), we found that the *Alcolapia* group, comprised of species adapted to extreme soda-lake environments (Trewavas 1983), is nested within the *Oreochromis*, forming a clade with *O. esculentus* and *O. amphimelas*. This supports the conclusions of Ford et al. (2019) that *Alcolapia* is best considered as a subgenus within *Oreochromis*. We consistently found *O. amphimelas* to be sister to *A. grahami.* This would suggest a single origin of tolerance of soda-lake conditions, which is present in *O. amphimelas*, albeit less extreme than *Alcolapia* (Trewavas 1983). However, unlike Ford et al., (2019), our analyses suggest that the species previously placed in the subgenus *Nyasalapia* (“Chambo”, *O. rukwaensis*, *O. chungruruensis*, *O. variabilis*, *O. malagarasi*, *O. macrochir*, and *O. karomo*) (Trewavas 1983) do form a monophyletic clade, with the exception of *O. chungruruensis*, which has a hybrid origin between the “Chambo” and the *O. shiranus—O. placidus* group.

Our study raises several taxonomic questions related to species classifications within the *Oreochromis*. Two species of the subgenus *Nyasalapia*, *O. squamipinnis*, and *O. karongae*, which are morphologically similar and endemic to Lake Malawi, comprise a single clade, but were not resolved as reciprocally monophyletic. Within the main lake, they have been differentiated largely on the basis of male breeding dress (Trewavas 1983), but populations in crater lakes have not been thoroughly studied and have just been provisionally identified on the basis of male color. The low number of specimens from Lake Malawi, as opposed to elsewhere in the catchment, prevents us from drawing clear conclusions about its populations. Our results suggest that the current taxonomic status of *O. shiranus* and *O. placidus* needs revision. Each of these species is presently split into two geographically separated subspecies (Trewavas 1983). Our results suggest that the current groupings are incorrect and should be replaced by a Lake Malawi/Shire/Zambezi taxon and a Chilwa/Chiuta/Ruvuma taxon, which better reflects historic and present river system connectivity. There are few, if any, morphological features supporting the current classification. We found that the *Oreochromis* population from the Mtera Reservoir did not cluster with *O. rukwaensis* sampled from Lake Rukwa. Instead, it was sister to a clade consisting of *O. rukwaensis, O. variabilis,* and *O. malagarasi.* This first genomic assessment of the population is consistent with the Mtera population representing a candidate new species, which we refer to as *O.* sp “mtera”. There was some apparent introgression between *O. rukwaensis* and *O. “*mtera”, perhaps explaining the morphological similarity that led to previous assumptions of their conspecific status (Genner et al. 2018).

### Widespread Ancestral Introgression

We found that there was widespread introgression across the *Oreochromis* phylogeny. The degree of introgression was only weakly predicted by phylogenetic distance or whether species occupy the same drainage basin in the modern day. This is, however, difficult to test given the highly correlated nature and lack of independence of introgression statistics, which are calculated on different subsets of four taxa across a phylogenetic tree. By identifying the species pairs with the highest *f*-branch scores, we accounted for this lack of independence, but may have lacked power to identify these influences. We highlight two notable instances of introgression between relatively phylogenetically divergent species, primarily between the “Chambo” and *O. shiranus* (likely resulting in *O. chungruruensis*), and between *O. niloticus* and *O. leucostictus.* These latter two species coexist in their indigenous habitat of the Albertine Rift lakes Edward, George, and Albert, and have been co-translocated to the same water bodies (e.g. Lake Victoria), yet are rarely found to hybridize in the modern day (Ciezarek et al. 2022; Geletu and Zhao 2023). These results suggest that the relatively low species-richness of *Oreochromis* tilapia, in contrast to the haplochromine cichlids, has not been a result of a lack of introgression. This is despite their similar distributions, habitat use, and life-history traits. Introgression has been demonstrated to fuel diversification in a wide taxonomic range of plant and animal species (Litsios and Salamin 2014; Stankowski and Streisfeld 2015; Marques et al. 2019; Slovák et al. 2023; Zhao et al. 2024). Further work on a wide range of organisms will be necessary to elucidate the conditions under which introgression drives adaptive radiations.

### Hybrid Speciation

Although introgression has not driven a major adaptive radiation in *Oreochromis*, it may have resulted in the origin of single species. We found that *O. chungruruensis* could have arisen as a result of hybrid speciation between “Chambo” and either *O. shiranus shiranus* or the wider *O. shiranus/O. placidus* group. Interestingly, *O. chungruruensis* is currently classified as a member of the subgenus *Nyasalapia* alongside *O. squamipinnis* and *O. karongae* (Trewavas 1983), which was supported by previous studies based on a handful of nuclear and mitochondrial markers (Ford et al. 2019). *O. chungruruensis* is endemic to Lake Kiungululu, an isolated crater lake within the northern sector of the Lake Malawi basin. Neither “Chambo” nor *O. shiranus* have been recorded in this lake, although “Chambo” and *O. shiranus shiranus* are found throughout the Malawi basin and co-occur in several crater lakes in the northern sector of the catchment (Kingiri, Ilamba, Itamba, and Ikapu). Indeed, our analysis finds two recent hybrids between “Chambo” and *O. shiranus shiranus* in Lake Itamba. The age of Lake Kiungululu is not known, although the nearby crater Lake Masoko has been estimated to be around 50,000 years old (Garcin et al. 2006), relatively soon before our estimated introgression ≤30,000 years ago. This may suggest a scenario where *O. chungruruensis* originated from hybridization between ancestral populations within the lake. Further detailed demographic analysis, based on deep sequencing of a larger number of individuals of each species, will be necessary to confirm this hybrid speciation as well as the exact timings and contributions of each parent population.

### Recent Hybridization Among *Oreochromis* Species

Within Tanzania, there has been a long history of translocations of *Oreochromis* species for capture fisheries, dating back to at least the 1950s, in addition to ongoing aquaculture translocations, leading to the widespread colonization by three species: *O. niloticus*, *O. leucostictus*, and *O. esculentus* (Shechonge et al. 2019). We note the limitations and potential confounding factors of hierarchical-clustering based analyses for detecting recent hybrids, such as genetic bottlenecks (Lawson et al. 2018), or hybrid speciation (as seen here in *O. chungruruensis*). However, our results demonstrate multiple ancestry components only in some individuals within populations, which would more likely reflect recent hybridization. Our results are also consistent with the long-documented interspecific hybridization between *Oreochromis* species based on SNP panels, mitochondrial data, and microsatellites (Geletu and Zhao 2023). Our study demonstrates that admixture between the invasive *O. niloticus, O. leucostictus*, and the native *O. urolepis*, previously recorded in Tanzania (Ciezarek et al. 2022) is widespread. Although we found no evidence of hybrids between the invasive *O. leucosticus* and *O. niloticus*, we did find five individuals which had ancestry components of over 0.1 (10%) for both species as well as *O. urolepis*. This suggests that there may be barriers preventing *O. leucostictus* and *O. niloticus* from directly hybridizing in the modern day, despite the ancestral introgression we inferred between them. Hybridization from either species with *O. urolepis* may however mediate gene flow between them. Previous studies, based on mitochondrial data have recorded introgression directly between the two species (Ndiwa et al. 2014), although Nyingi et al. (2009) found this introgression signal was not present in the nuclear genome. Furthermore, it is not clear whether there was thorough species sampling in these studies, raising the possibility of “ghost” introgression explaining these findings. Further work is necessary to determine whether there are genomic or behavioral barriers reducing the likelihood of *O. leucostictus* and *O. niloticus* hybridizing directly. Ongoing monitoring, and the generation of temporal datasets, will now be necessary to examine the potential risk of hybridization to both farmed and wild populations.

Interestingly, the Lake Chala Bandia individual appears to be a case of hybridization between two species not native to the lake (*O. urolepis* and *O. rukwaensis*), plausibly in farm ponds prior to its introduction to the lake. Previous studies on Bandia have suggested some individuals clustered with *O. urolepis*, but with a high degree of diversity in both mitochondrial DNA and morphology (Dieleman et al. 2019; Moser et al. 2019). This is consistent with our finding of hybrid origin. Our study is, however, based on only a single Bandia individual, and so more detailed population genomic analysis would be welcome.

### Concluding Remarks

Several caveats need to be considered when assessing our findings. Critically, our study did not include every existing species or population of *Oreochromis* found across the globe. These missing or “ghost” taxa, in addition to extinct populations of *Oreochromis* may have significantly misled analyses of ancestral introgression (Tricou et al. 2022a, 2022b). Extensive sampling of tilapia at both the species and population levels across Africa and the Middle East will be necessary to assess this, in addition to the development of bioinformatic tools which can accurately infer phylogenetic networks with large datasets, including genome-scale data and many individuals. Ghost taxa may have influenced the identification of recent hybrids, particularly in water bodies where *Oreochromis* was less thoroughly sampled. We also note the relatively low depth of sequencing we used. Deeper sequencing in the future will enable more detailed and confident demographic analyses of these populations, and more precise quantification of the extent of gene flow.

Our results highlight the complex relationship between farmed animals and their wild relatives. The diversity that exists within the wild may be used to enhance that of farmed populations by selective breeding (Bentsen et al. 2017). Ongoing genomic monitoring and assessment will be necessary to examine the true extent of hybridization, its evolution over time, and to what extent it influences the diversity existing in wild populations. This will be necessary to meet both food security and conservation outcomes.

## Supplementary Material

msae116_Supplementary_Data

## Data Availability

All raw-read data generated is available in the European Nucleotide Archive (project PRJEB77041). All inferred phylogenetic trees and their underlying datasets are available in Dryad (https://doi.org/10.5061/dryad.p2ngf1w0c). All custom R and Python scripts are available in Zenodo (https://doi.org/10.5281/zenodo.12549228). Any further data is available upon request.
